# Labellable Phylogenetic Networks

**DOI:** 10.1007/s11538-023-01157-0

**Published:** 2023-04-25

**Authors:** Andrew Francis, Mike Steel

**Affiliations:** 1 Centre for Research in Mathematics and Data Science, Western Sydney University, Penrith, Australia; 2 Biomathematics Research Centre, Canterbury University, Christchurch, New Zealand

**Keywords:** Phylogenetic network, Tree-based, Orchard, Encoding

## Abstract

Phylogenetic networks are mathematical representations of evolutionary history that are able to capture both tree-like evolutionary processes (speciations) and non-tree-like ‘reticulate’ processes such as hybridization or horizontal gene transfer. The additional complexity that comes with this capacity, however, makes networks harder to infer from data, and more complicated to work with as mathematical objects. In this paper, we define a new, large class of phylogenetic networks, that we call labellable, and show that they are in bijection with the set of ‘expanding covers’ of finite sets. This correspondence is a generalisation of the encoding of phylogenetic forests by partitions of finite sets. Labellable networks can be characterised by a simple combinatorial condition, and we describe the relationship between this large class and other commonly studied classes. Furthermore, we show that all phylogenetic networks have a quotient network that is labellable.

## Introduction

The problem of describing the way that a set of organisms are related through evolution is usually answered by presenting a phylogenetic tree or network in which the leaves are labelled by the set of species. These are directed acyclic graphs with a single root that is a common ancestor of the set of species. The internal vertices represent historical events that include speciation, in which a vertex has out-degree two or greater, or some form of reticulation (hybridization or horizontal gene transfer (HGT)), in which a vertex has in-degree two or greater. Phylogenetic trees are a special case of networks in which all internal vertices have in-degree 1, and thus the only event represented by the vertices is speciation.

Manipulating either trees or networks mathematically is important because many methods for determining a tree or network require searching to find an optimum, and they often involve choosing random trees or networks. To make this possible, encoding such graphs in other mathematical formats is often important computationally. For instance, a classical encoding of trees is the Newick format, which records clusters (descendents of internal vertices) in a structured, in-line notation (used in Felsenstein (1989)), and there are encodings using sequences of integers (Bandelt and Dress (1986); James Rohlf (1983)). In networks, classes can be encoded by specific substructures, such as ‘trinets’ (for tree-child networks) (van Iersel and Moulton 2014), or additional structures such as the use of ‘circular’ permutations for a class of *unrooted* phylogenetic networks (Francis et al. 2023). A sub-class of (unlabelled) tree-child networks on *n* leaves can also be encoded by words of a certain type over an alphabet of size *n* (Fuchs et al. 2021).

In this paper, we show that a large class of rooted phylogenetic networks is encoded by certain covers of finite sets. This specifically generalises the encodings for phylogenetic trees and forests that were given for binary trees by Diaconis and Holmes (1998), and for general phylogenetic forests more recently (Francis and Jarvis 2022).

We call this encodable class *labellable* phylogenetic networks, because they have the property that their internal vertices can be deterministically labelled via an algorithm based on an approach developed for trees (Erdős and Székely 1989).

There are many classes of phylogenetic network, and new ones are regularly defined. The reason is that inferring networks is a difficult problem, partly because the space of all networks is too large, and partly because many networks have features that are unlikely to be inferable from data or that make them difficult to work with mathematically. The class of labellable networks is a new and large class, as we will show in Sect. 6. Its most important property—that its elements are in bijection with the set of *expanding covers* (we define this later)—means that these networks can be studied using an elementary combinatorial (set-theoretic) structure. Surprisingly, we are able to show that every phylogenetic network has a labellable quotient.

The paper begins with a background section giving the formal definitions and recalling some relevant results from earlier work. Section 3 describes an algorithm for labelling the internal vertices of a network, and defines a labellable network as one for which this algorithm is well defined. It also provides a structural characterisation of labellable networks in Theorem 3.3. The next section (Sect. 4) gives the connection to covers and proves that the set of labellable networks is in bijection with the set of ‘expanding covers’ (Theorem 4.4). This correspondence is made explicit for non-degenerate networks in Sect. 5. In Sect. 6, we then consider the class of labellable networks and its relationships with other well-known classes, such as tree-based networks, tree-child networks, orchard networks, and others. We show that the class of labellable networks contains all orchard networks, and hence all tree-child and normal networks, but it does not contain (and is not contained in) the class of tree-based networks. We characterise exactly when a binary tree-based network is also labellable, in Theorem 6.3. Figure 6 shows the relationships among some of these classes. Finally, in Sect. 7, we show that by defining an equivalence relation on the vertices in any phylogenetic network, we can form a quotient network that is labellable. We call the quotient the *derived* network and briefly discuss the relationship of this quotient to the normalisation map that was recently defined (Francis et al. 2021). We end with a discussion that highlights some questions for further research.

## Background

### Phylogenetic Trees and Networks

A *(rooted) phylogenetic network* on *n* leaves is a directed acyclic graph $N=(V,A)$ with vertex set *V* and directed edge set *A*, that has a single root (a vertex with in-degree 0) and *n* leaves (vertices of out-degree 0 and in-degree 1) labelled by $[n]:=\{1,\ldots ,n\}$. Vertices that are not leaves or the root are called *internal*. The set of rooted phylogenetic networks on *n* leaves is denoted $\mathcal {R}\negthinspace \mathcal {P}\negthinspace \mathcal {N}_n$. In this paper, all networks are rooted, so we will drop the word ‘rooted’.

This is a more general definition than sometimes used, in that it allows the internal vertices to have any nonzero in-degree and any nonzero out-degree. A *phylogenetic tree* is a phylogenetic network in which all internal vertices have in-degree 1.

We draw a phylogenetic network with its single root at the top, its leaves at the bottom, and directed edges drawn with direction running down the page. The vertices of a phylogenetic network are called *tree vertices* if they have in-degree 1 (including leaves) and are called reticulate vertices (or reticulations) if they have in-degree greater than 1.

We say an internal vertex is *degenerate* if it either has both in-degree and out-degree equal to 1, or both in-degree and out-degree strictly greater than 1 (the former case are *degenerate tree vertices*; the latter are *degenerate reticulations*). A network is called *non-degenerate* if it contains no degenerate vertices.

A phylogenetic network is *binary* if the root has out-degree 2, and all internal vertices have total degree 3 (in-degree 2 and out-degree 1, or vice versa). A *non-binary* phylogenetic network is one that is not necessarily binary.

Let $c_N(x)$ denote the set of *children* of *x* in *N*, that is, the set of vertices *y* in *N* for which there is an edge from *x* to *y*. Let $p_N(x)$ denote the set of *parents* of *x* in *N*, that is, the set of vertices *w* in *N* for which there is an edge from *w* to *x*.

Vertices in a phylogenetic network are *siblings* if they share the same parent. A *cherry* in a phylogenetic network is a pair of leaves that are siblings. A *reticulated cherry* is a pair of leaves for which the parent of one is a sibling of the other.

### Trees, Forests, and Labellings

**Fig. 1:**
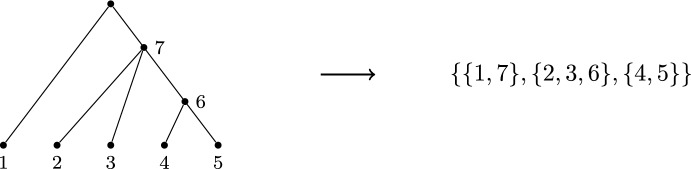
A tree that has been labelled according to the algorithm in Erdős and Székely (1989), and its corresponding partition for which the sets are sets of sibling vertices. This labelling algorithm is a special case of that given later for networks, in Algorithm 1

Phylogenetic trees and forests (collections of trees in which the leaves partition the set $\{1, \ldots , n\}$) have been shown to correspond to various partitions of finite sets. The basis for this correspondence is a labelling algorithm that, given a phylogenetic tree with leaves labelled $1,\dots ,n$, assigns labels to the internal vertices of the tree in increasing order from $n+1$ as far as we are aware, this algorithm first appeared in Erdős and Székely (1989)). In the case of binary trees (those in which the internal vertices have out-degree 2), there are $2n-2$ labels in total, and a correspondence with perfect matchings follows by forming pairs of the labels of sibling vertices (Diaconis and Holmes 1998). In the case of non-binary trees and phylogenetic forests, the same process of forming sets of sibling vertices gives a correspondence between the set of phylogenetic forests and all partitions of finite sets (Francis and Jarvis 2022) (see Fig. 1). Note, an alternative labelling algorithm for a class of forests that include degree-two vertices has been given in Erdős (1993), and this gives rise to a different correspondence than that of Francis and Jarvis (2022).

In the next section, we show how this labelling algorithm for trees and forests can be extended to some, but not all, phylogenetic networks. Those for which it can be extended we call ‘labellable’ networks.

## Labellable Networks

The labelling algorithm for binary trees given by Erdős and Székely (1989) and Diaconis and Holmes (1998), and extended to non-binary trees and forests in Francis and Jarvis (2022), takes a tree or forest with leaves labelled by [*n*], and progressively labels the internal vertices in sequence until all except the root are labelled. At each step, the algorithm chooses a new vertex to label from those for which the children are all labelled, by choosing the one whose children have the lowest label. Note, with trees and forests, the sets of the children of vertices are disjoint.

In networks, the sets of the children of internal vertices are not, in general, disjoint, and it is necessary to define the following partial order (the lexicographic order on sets) to facilitate the labelling algorithm:

$$\begin{aligned} A\prec B\quad \text {if}\quad A\subseteq B\quad \text {or}\quad \min \left( A\setminus (A\cap B)\right) <\min \left( B\setminus (A\cap B)\right) .\end{aligned}$$

Note that this order reduces to the order used for labelling internal vertices in phylogenetic forests (in Algorithm 1 of Francis and Jarvis (2022)).

**Figure:**
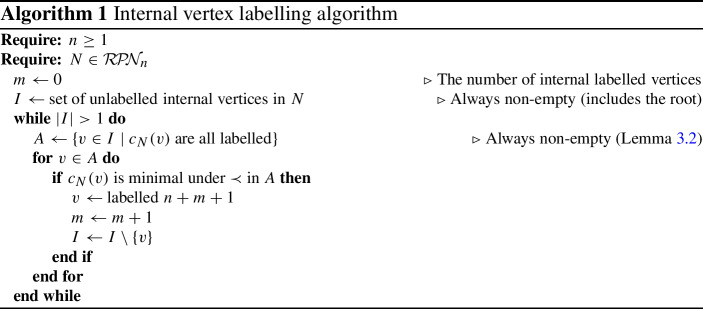
Internal vertex labelling algorithm

Algorithm 1 begins with a network in which the leaves are labelled by $[n]=\{1,\ldots , n\}$ with the usual total ordering. At each step, the algorithm takes a network that has been partially labelled, chooses the minimal set of labelled sibling vertices (with respect to $\prec$) for which the parent is not labelled, and assigns the next available integer to the parent. The output is a network in which the (non-root) internal vertices are labelled by the elements of $\{n+1, \ldots , n+(\#\text {internal vertices}) - 1\}$.

Algorithm 1 is well defined for a network *N* as long as (a) it is always possible to find a set of sibling vertices for which the parent is unlabelled, and (b) each set of labelled siblings has only one parent. These are essentially existence and uniqueness conditions.

### Definition 3.1

A *labellable* network is one for which Algorithm 1 is well defined.

**Fig. 2:**
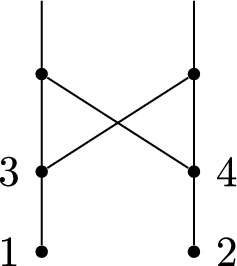
A network substructure that is not labellable, since the vertices labelled 3 and 4 share the same set of parents

Although we can show that (a) holds for all phylogenetic networks (Lemma 3.2), it is easy for (b) to fail (see Fig. 2).

### Lemma 3.2

Let *N* be a network with some vertices labelled, including all leaves. If *N* has any unlabelled vertices, then it contains at least one unlabelled vertex for which the children are all labelled.

### Proof

For a vertex *v* in *N*, define *d*(*v*) to be the length of the longest path from *v* to a leaf. This is well defined, since *N* is finite and acyclic.

For *v* an unlabelled vertex in *N*, if there is an unlabelled vertex $v'$ on a path from *v* to a leaf, then $d(v')<d(v)$. However, *d* is bounded below by 1 for any unlabelled vertex (since the leaves themselves are already labelled). Therefore, repeating this process will eventually find an unlabelled vertex *u* for which all the vertices on the paths from *u* to a leaf are labelled. In particular, all of its children are labelled, as required. $\square$

### Theorem 3.3

A network *N* is labellable if and only if $c_N(x)\ne c_N(y)$ for all non-leaf vertices $x\ne y$ in *N*. That is, *N* is labellable if and only if $c_N$ is one-to-one.

### Proof

Lemma 3.2 shows that for any phylogenetic network, the labelling algorithm will always find a set of labelled vertices for which the parent is unlabelled. Let *U* be the set of such unlabelled vertices that are the parents of labelled children. For the algorithm to be well defined, it must be possible to use $\prec$ to place an order on the set *U*, via their sets of children.

The forward direction is immediate: if a network is labellable, then it cannot have a pair of distinct vertices with the same set of children, since those distinct vertices would then not be able to be ordered.

Suppose, for the reverse direction, that the sets of children of each vertex in *N* are distinct. In this case, any pair of unlabelled vertices with labelled children has distinct sets of children, and these can be ordered by $\prec$, thus allowing *N* to be labelled. $\square$

## Covers and Labellable Networks

As with trees and forests, the labelling of non-root vertices in a network gives rise to a set of subsets of integers, namely the sets of sibling vertices (those sharing a common parent).

There are two differences between this set-up for networks and that in earlier work on forests. First, singleton sets may represent the children of reticulations in the networks, or, indeed, degree-two internal vertices, instead of the roots of trees. Second, the sets are not disjoint, because an integer labelling a reticulate vertex will have two sets of siblings. Consequently, the sets of sibling vertices from a labellable network are not a partition of [*m*]. Instead, they are a ‘cover’ of [*m*], where a *cover* of [*m*] refers to a set of non-empty subsets of [*m*] for which the union is [*m*]. Trivially, since a cover is a set, all of its member sets are distinct. The sets in a cover ${\mathcal C}$ have a total order $\prec$ as defined above. We will denote the number of sets in ${\mathcal C}$ by $\vert {\mathcal C}\vert$.

**Fig. 3:**
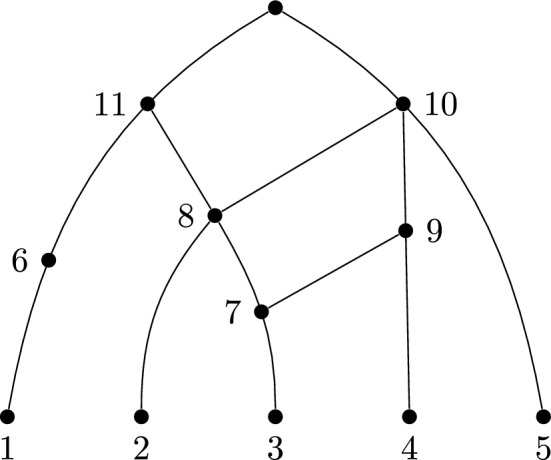
A labelled, degenerate phylogenetic network. This network has the associated cover $\{\{1\},\{3\},\{2,7\},\{4,7\},\{5,8,9\},\{6,8\}$, $\{10,11\}\}$

We will call the set of the sets of sibling vertices from a phylogenetic network *N* *the cover associated with N*. An example is given in Fig. 3. Note that repeated labels in a cover from a network correspond to reticulations.

Lemma 4.1 is an analogue of a similar result for forests in the proof of (Francis and Jarvis (2022) Theorem 6.5).

### Lemma 4.1

If ${\mathcal C}$ is a cover of [*m*] associated with a phylogenetic network on *n* leaves, then $n=m-\vert {\mathcal C}\vert +1$.

### Proof

We can count the number of vertices in the network in two ways. If the number of sets in ${\mathcal C}$ is $\vert {\mathcal C}\vert$, then there are $\vert {\mathcal C}\vert$ non-leaf vertices in *N* and, consequently, $n+\vert {\mathcal C}\vert$ vertices overall. On the other hand, all vertices in the network are labelled by a unique element of [*m*], except the root. So there are $m+1$ vertices in *N*. Thus, $n+\vert {\mathcal C}\vert =m+1$ and so $n=m-\vert {\mathcal C}\vert +1$ as required. $\square$

If a cover ${\mathcal C}$ of [*m*] comes from a network, it also satisfies the properties in the following lemma:

### Lemma 4.2

If a cover ${\mathcal C}$ comes from a labellable network *N* on *n* leaves, then:

1. The elements of $\{1,\dots ,n\}$ are not repeated in ${\mathcal C}$; and
2. For each $i=1,\dots , \vert {\mathcal C}\vert$, ${\mathcal C}$ contains at least *i* subsets of $[n+i-1]$.

### Proof

Suppose ${\mathcal C}$ is the cover associated with the labellable network *N*.

The leaves, labelled $1,\dots ,n$, have in-degree 1, and thus each has precisely one parent, and they are all members of precisely one set of siblings. Thus, the labels of leaves are not repeated in ${\mathcal C}$, proving (1).

Claim (2) can be proved by induction on *i*. If *N* is labellable, then, in the first step, there must be at least one set in ${\mathcal C}$ that is wholly contained in $\{1,\dots ,n\}$. The minimal such set with respect to $\prec$ is denoted $X_1$, and the label $n+1$ is added to the vertex in *N* whose children are labelled by $X_1$.

Suppose that for each $i\le k$, there are at least *i* subsets of $\{1,\dots ,n+i-1\}$ in ${\mathcal C}$. Here, at each step of the labelling algorithm up to and including step *k*, a new vertex is labelled for which the children are labelled by a set we denote $X_i$; in particular, at step *k*, the label $n+k$ is added to a previously unlabelled vertex in *N* for which the children are labelled by $X_k$.

Since *N* is labellable, after the *k*’th step, there are $n+k$ labelled vertices and a set of labelled vertices *Y* for which the parent is unlabelled. This set *Y* is distinct from $X_1,\dots ,X_k$, since the sibling vertices labelled by those sets all have their parents labelled by steps up to step *k*. Thus, we have $X_{k+1}:=Y$, which is a subset of $\{1,\dots ,n+k-1,n+k\}$, and we have $k+1$ sets in ${\mathcal C}$ that are subsets of $\{1,\dots ,n+(k+1)-1\}$, as required. $\square$

### Definition 4.3

A cover satisfying the conditions in Lemma 4.2 is called an *expanding cover*.

### Theorem 4.4

The set of labellable phylogenetic networks is in bijection with the set of expanding covers.

### Proof

Lemma 4.2 shows that each labellable network gives an expanding cover.

For the reverse direction, if a cover is expanding, then a network can be deterministically constructed from the cover, using the following algorithm.

**Figure:**
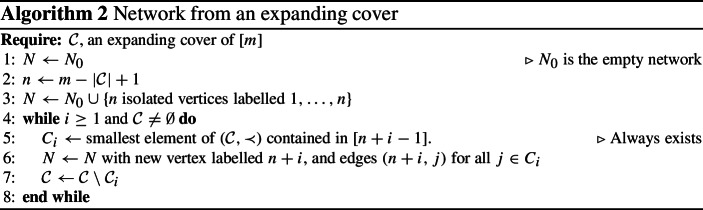
Network from an expanding cover

Note that in the intermediate stages, the graph is not necessarily a phylogenetic network because it may not be connected or it may have several vertices of in-degree 0.

This algorithm is well defined because of the properties of expanding covers—there will always be a set at the first step of the while loop (Line 5) to choose—and it will terminate because ${\mathcal C}$ is finite and is reducing in cardinality by 1 with each iteration of the while loop at Line 7. It will output a phylogenetic network because, with the exception of a single unlabelled vertex, every vertex has a label and every labelled vertex is in a set in ${\mathcal C}$ and thus has a parent. The single unlabelled vertex is the unique root, which has in-degree 0. $\square$

### Example 4.5

Consider the cover ${\mathcal C}=\{\{2\},\{5\},\{1,6\},\{4,8\},\{3,6,9\},\{10\}$, $\{7,11\}$, $\{8,12\}\}$ of [12], with $\vert {\mathcal C}\vert =8$. If this corresponds to a network, the network must have $n=12-8+1=5$, by Lemma 4.1.

It is expanding because it has two subsets of $[n]=[5]$ (the definition of an expanding cover requires at least one), three subsets of [6] (which needs at least two), three subsets of [7] (which needs three), four subsets of [8], five subsets of [9], six subsets of [10], seven subsets of [11], and eight subsets of [12].

**Fig. 4:**
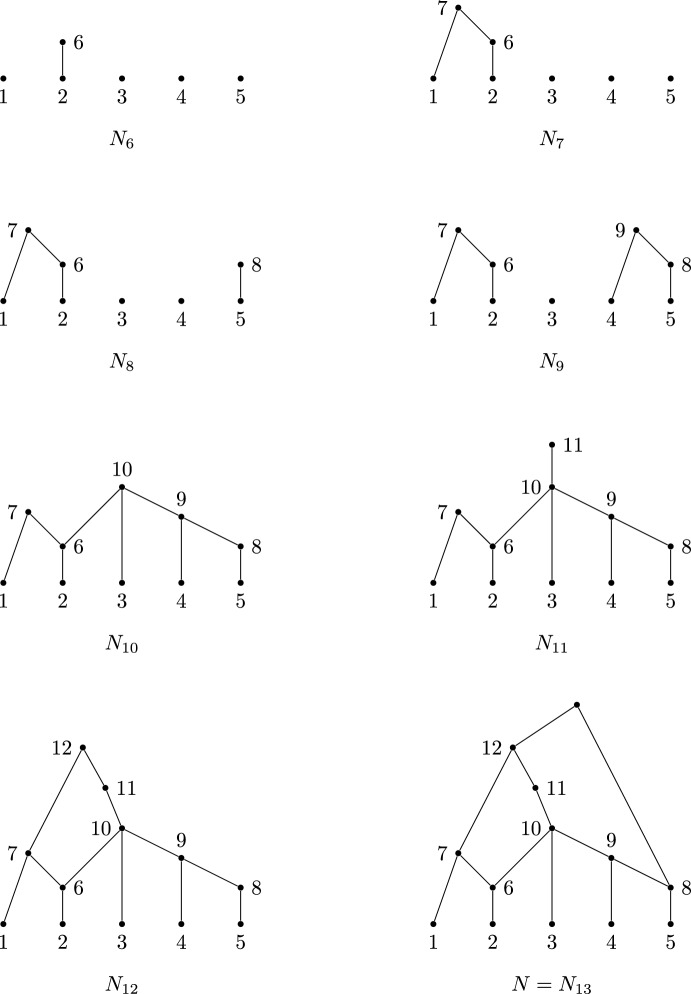
Construction of a network from the expanding cover ${\mathcal C}=\{\{2\},\{5\},\{1,6\},\{4,8\},\{3,6,9\},\{10\},\{7,11\},\{8,12\}\}$ described in Example 4.5. The first step, consisting of five isolated vertices, is omitted. Note that the labels of the reticulate vertices (6 and 8) appear twice in ${\mathcal C}$

See Fig. 4 for an illustration of the network constructed from this cover by Algorithm 2.

The set giving rise to the vertex label *i* is determined by its position in an ordering of the sets in ${\mathcal C}$. This ordering will use $\prec$ and is defined by the following algorithm:

1. For $$i=1,\dots ,\vert {\mathcal C}\vert $$, do:
  - $C_i$ is the minimal set in $({\mathcal C},\prec )$ contained in $[n+i-1]$.
  - Set $${\mathcal C}={\mathcal C}\setminus C_i$$.
2. Output sequence $$C_1,\dots ,C_{\vert {\mathcal C}\vert }.$$

Because this is the order in which sets in a cover are used to assign new labels to the vertices in the labelling algorithm (Alg. 1), we call it the ‘labelling order’ for a cover.

## Covers and Non-degenerate Networks

The constraint that networks must be non-degenerate requires an associated constraint on the corresponding set of covers. Non-degeneracy is equivalent to the condition that each internal vertex has either in-degree 1 or out-degree 1, but not both.

Let *N* be a non-degenerate labellable network with cover ${\mathcal C}$. In terms of ${\mathcal C}$, the in-degree of vertex *i* in ${\mathcal C}$ is precisely the number of sets in ${\mathcal C}$ for which *i* is an element, whereas the out-degree of *i* is the size of the set in ${\mathcal C}$ that gave rise to it. If *N* is non-degenerate, there must be corresponding constraints on the cover ${\mathcal C}$.

With the sequence on the sets in ${\mathcal C}$ given by the labelling order, we now have the following two-part ‘order condition’ for each $i\ge 1$:

- If $\vert C_i\vert >1$, then $(n+i)$ appears *at most once* in ${\mathcal C}$; and
- If $\vert C_i\vert =1$, then $(n+i)$ appears *more than once* in ${\mathcal C}$.

### Theorem 5.1

The set of non-degenerate phylogenetic networks are in bijection with the set of expanding covers satisfying the order condition.

### Proof

For any labellable phylogenetic network, we have a unique expanding cover, according to Theorem 4.4. If that network is non-degenerate, each vertex of out-degree greater than one must have in-degree equal to one, and each vertex of out-degree exactly one must have in-degree strictly greater than one.

If a non-leaf vertex in a labellable non-degenerate network *N* has the label *k*, then $k=n+i$ for some $i\ge 1$, since the leaves have labels 1 to *n*. This indicates that the label *k* was added by the *i*’th set $C_i$ in the labelling order on the expanding cover ${\mathcal C}$ that corresponds to *N*.

If the set $C_i$ has cardinality one, then the vertex labelled *k* has out-degree one, since its children are the vertices for which the labels are in $C_i$. Since *N* is non-degenerate, the in-degree of the vertex labelled *k* must be strictly greater than one. This implies that *k* has more than one parent and, therefore, more than one set of siblings. That is, *k* appears in more than one set in ${\mathcal C}$, as required.

On the other hand, if $C_i$ has cardinality strictly greater than one, then *k* has out-degree $\vert C_i\vert >1$, and since *N* is non-degenerate, it must have in-degree one. This is not possible if *k* appears more than once in ${\mathcal C}$, so it must appear just once, as required.

In the reverse direction, each expanding cover corresponds to a unique phylogenetic network by Theorem 4.4. If, in addition, the cover satisfies the order condition, then (a) each vertex label that arises from a set $C_i$ of cardinality greater than one appears just once, and (b) each vertex label that arises from a set $C_i$ of cardinality exactly one appears more than once. These constraints, respectively, force the vertex to have in-degree one and out-degree more than one, or in-degree more than one and out-degree one. In other words, the vertex is non-degenerate, and so the network, as a whole, is non-degenerate. $\square$

The order condition feels somewhat unsatisfying because it is not a passive property of the cover, but requires an additional order to be placed on it (the labelling order), and a check to be performed algorithmically. By contrast, the conditions for expanding covers are a static check for each $i=1,\dots ,\vert {\mathcal C}\vert$. However, it is ‘easy’ to check that the order condition holds for a given cover. The construction of the order on ${\mathcal C}$ is linear in $\vert {\mathcal C}\vert$, and the check of the order condition itself seems to be quadratic in $\vert {\mathcal C}\vert$, because the check is done for each $i=1,\dots ,\vert {\mathcal C}\vert$, and for each check, membership is tested for each set in ${\mathcal C}$. In other words, the whole process is, at worst, cubic in complexity.

### Example 5.2

Recall that the cover used in Ex. 4.5 was

$$\begin{aligned} {\mathcal C}=\{\{2\},\{5\},\{1,6\},\{4,8\},\{3,6,9\},\{10\},\{7,11\},\{8,12\}\}.\end{aligned}$$

The labelling order on ${\mathcal C}$ is as used in the network construction shown in Fig. 4 (by design), namely $(\{2\},\{1,6\},\{5\},\{4,8\},\{3,6,9\},\{10\},\{7,11\},\{8,12\})$. That is, $C_1=\{2\}$, $C_2=\{1,6\}$, and so on, with $n=5$. To check the order condition here, we need to look at sets of size 1 and those of size $>1$. The three of size 1 are $C_1=\{2\}$, $C_3=\{5\}$, and $C_6=\{10\}$. The order condition would therefore require $6=n+1$, $8=n+3$, and $11=n+6$ to appear more than once in ${\mathcal C}$. This is satisfied for 6 and 8, but not 11. This is seen in the resulting network, as 11 labels a degenerate vertex of in-degree and out-degree 1.

The sets of size $>1$ are $C_2,C_4,C_5,C_7$, and $C_8$, and all of $n+i$ for $i=2,4,5,7,8$ appear at most once in ${\mathcal C}$ and so do not give degenerate vertices. Note that the last of these, $13=5+8$, does not appear at all, as the vertex added by that set is the last, which is the unlabelled root.

## Labellable Networks and Other Familiar Classes

The property of being labellable defines a class of networks, so it is natural to ask whether it is, in fact, one of the known classes and, if not, how it relates to the many well-studied classes of phylogenetic networks. For an overview of the many known classes, we refer the reader to [Steel (2016) Chapter 10], or Kong et al. (2022).

Most of the well-studied classes are defined without permitting degenerate vertices (for instance, orchard networks have been defined in the non-binary case (van Iersel et al. 2022), but not with degenerate vertices), so many results in this section are restricted to the non-degenerate case, whether binary or not.

Of the many classes previously defined, most are contained in the class of tree-based networks (Francis and Steel 2015)—which are networks that have a spanning tree for which the leaves are those of the network—so we will first ask whether all labellable networks are tree-based.

It turns out that not all tree-based networks are labellable, because the property of being labellable excludes substructures such as that in Fig. 2, which are allowable in tree-based networks, as the following result makes clear.

### Corollary 6.1

(Corollary to Theorem 3.3) If *N* is a non-degenerate tree-based network, then *N* is labellable if and only if $c_N(x) \ne c_N(y)$ for all tree vertices $x \ne y$ in *N*.

### Proof

By Theorem 3.3, it suffices to show that for any non-degenerate tree-based network *N*, the equality $c_N(x) = c_N(y)$ cannot hold if one or both of *x* and *y* is reticulate. First suppose that *x* and *y* are both reticulate vertices. By the non-degenerate condition, *x* and *y* each have exactly one (identical) child *z*, and again by the non-degenerate condition, *z* is a reticulate vertex. However, this configuration of reticulation vertices (two reticulations with a reticulate child) cannot exist in a tree-based network (by the antichain-to-leaf property described in Francis and Steel (2015)). Alternatively, suppose that *x* is a reticulate vertex and *y* is a tree vertex. Again, by the non-degenerate condition, *x* has a single child, while *y* has at least two children and so $c_N(x) \ne c_N(y).$ $\square$

### Remark 6.2

A further result is that if *N* is semiresolved (i.e. every tree vertex has out-degree 2), then *N* is labellable if *N* is stable (this follows from Theorem1 of Huber et al. (2016), where the notion of ‘stable’ is defined).

In the case of *binary* tree-based networks, a tighter characterisation of those that are labellable is possible, as follows.

### Theorem 6.3

A binary tree-based network is labellable if and only if it has the property that $p_N(x) \ne p_N(y)$ for each pair of reticulate vertices *x*, *y*.

### Proof

Let *N* be a binary tree-based network.

Suppose that $p_N(x)=p_N(y)$ for a pair of reticulate vertices. Since *N* is binary, *x* and *y* both have the same two parents (say *u*, *v*), and so (again since *N* is binary) $c_N(u)=c_N(v)$. Hence, by Theorem 3.3, *N* is not labellable.

Conversely, suppose that *N* is not labellable. Theorem 3.3 implies the existence of vertices *u*, *v* with $c_N(u)=c_N(v)$. Since *N* is binary, this implies that either (a) $c_N(u)=c_N(v) =\{x,y\}$ for a pair of reticulate vertices *x*, *y*, so $p_N(x)=p_N(y)$; or (b) $c_N(u)=c_N(v) =\{x\}$. However, this second case cannot occur in a binary tree-based network, since it implies that *u*, *v* and *x* are three reticulations with the arcs (*u*, *x*) and (*v*, *x*), which is impossible in a binary tree-based network. Thus, $p_N(x)=p_N(y)$ for a pair of reticulate vertices. $\square$

**Fig. 5:**
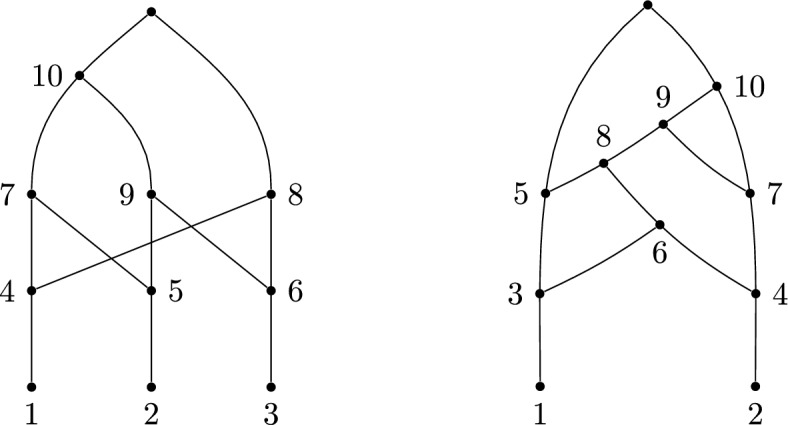
Left: A labellable network that is not orchard. It can be seen to not be orchard because it does not have any cherries or reticulated cherries. Right: A labellable network that is not tree-based. This can be seen because it has a substructure called a zig-zag path, which prevents a network from being tree-based (Zhang 2016), namely between the reticulate vertices 5 and 7 along the path $5-3-6-4-7$

Although not all tree-based networks are labellable, it is also true that not all labellable networks are tree-based: the network shown at the right of Fig. 5 can be labelled, but is not tree-based.

In summary, the classes of labellable and tree-based networks are not nested within each other.

**Fig. 6:**
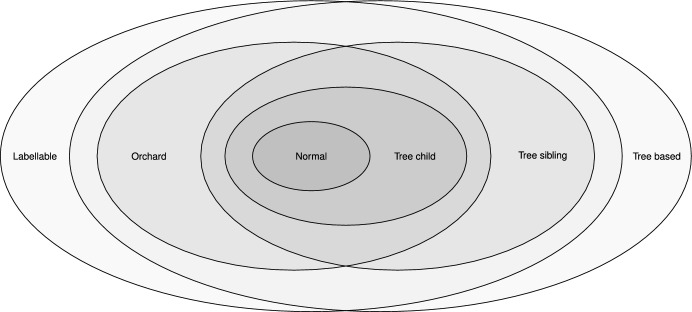
Relationships between labellable networks and some other classes. This figure applies for non-degenerate networks (non-binary networks permitted)

In the remainder of this section we show that some other large classes that sit inside the tree-based networks—the orchard networks and tree-sibling networks—are also labellable. Thus, they sit in the intersection of the tree-based and labellable classes (see Fig. 6). Furthermore, other prominent classes of network are contained inside the intersection of orchard and tree-sibling networks. These include tree-child networks (Janssen and Murakami 2021), and normal networks, which are tree-child (as also shown in Fig. 6).

*Orchard networks* are non-degenerate, rooted phylogenetic networks that have the defining property that they can be reduced to a single point by a series of cherry reductions and reticulated cherry reductions. These reductions replace a cherry or reticulated cherry with a simpler structure, progressively reducing the network. See (Erdős et al. 2019) for the original definition, and (van Iersel et al. 2022) for the extension to the non-binary case and, in particular, a result we use in the proof below.

### Theorem 6.4

Orchard networks are labellable.

### Proof

Suppose that *N* is an orchard network, but is not labellable (we will derive a contradiction). Since every orchard network (binary or non-binary) is tree-based [by Corollary 4.5 of van Iersel et al. (2021)], Corollary 6.1 implies that *N* has a pair of tree vertices $u_1, u_2$ with $c_N(u_1)=c_N(u_2)$. Since $u_1$ and $u_2$ are tree vertices (and *N* is non-degenerate), this shared set of children has size at least 2.

By Theorem 2 of van Iersel et al. (2022), a non-degenerate network *N* is orchard if and only if some binary resolution of *N* (or *N* itself, if *N* is binary) has a ‘HGT-consistent labelling’, namely, a map $t: V \rightarrow {\mathbb R}$ that satisfies the following three conditions:

1. For all arcs (*u*, *v*), $t(u) \le t(v)$ and equality is allowed only if *v* is a reticulation.
2. For each internal vertex *u*, there is a child *v* of *u* such that $t(u) < t(v)$.
3. For each reticulation *r* with parents *u* and *v*, exactly one of $t(u) = t(r)$ and $t(v) =t(r)$ holds.

The vertices $u_1$ and $u_2$ with their shared set of children (of size $k \ge 2$) provide an obstruction to a HGT-consistent labelling of any binary resolution of *N*, as follows.

Consider a network $N_k$ consisting of: a root whose children are $u_1$ and $u_2$; their shared *k* children $v_1,\dots ,v_k$; and *k* leaves, one descending from each of the $v_i$. This network is not orchard, because it does not have any cherries or reticulated cherries, and this remains true for any binary resolution of $N_k$. Therefore, there is no HGT-consistent labelling of any binary resolution of $N_k$.

Notice that $N_k$ contains a copy of $K_{2,k}$ (the complete bipartite directed graph on vertex sets of size 2 and *k*), and the only non-binary vertices in $N_k$ are the two parent vertices $u_1$ and $u_2$. Now, if a network $N'$ contains a copy of $K_{2,k}$ then every binary resolution of $N'$ fails to have a HGT-consistent labelling (for otherwise, if one did exist, then that resolution and HGT-consistent labelling could also be applied to $N_k$). Since *N* contains a copy of $K_{2,k}$ (via its vertices $u_1$ and $u_2$ and their shared sets of children), *N* has no binary resolution with a HGT-consistent labelling, and so is not an orchard network. $\square$

### Corollary 6.5

If *N* is an orchard network, then it does not contain any two vertices that have the same set of children.

### Proof

Since *N* is orchard it is labellable, according to Theorem 6.4, and the result follows from the characterisation in Theorem 3.3. $\square$

Note, there are labellable networks that are not orchard, such as that in Fig. 5.

Theorem 6.4 also implies that tree-child networks (Cardona et al. 2008) are labellable (since they are a subset of the orchard networks), but this can also be proved directly by looking at the sets of the children of vertices, as follows.

Recall that a vertex *v* in a network is *visible* if there is a leaf $i \in [n]$ for which every path in *N* from the root to leaf *i* passes through *v*.

### Lemma 6.6

In any phylogenetic network *N*, if $c_N(u)=c_N(v)$, then neither *u* nor *v* is visible.

### Proof

Suppose that $c_N(u)=c_N(v)$ and that *u* is visible. In this case, there is a leaf $i \in [n]$ for which every path *P* from $\rho$ to *i* passes through *u*. Since *P* must also pass through one of the children of *u* (say *x*), let $P'$ be any path from $\rho$ to *v*, and extend this path by adding the edge (*v*, *x*) followed by the path used by *P* from *x* to *i*. The path $P'$ cannot pass through *u*, because the children of *u* and *v* are identical: if it passed through *u* either before or after passing *v*, that would force a directed cycle in the network. This extended path is therefore a path from $\rho$ to *i* that avoids *u*, contradicting the assumption that *u* is visible. $\square$

Note that a network *N* is tree-child if and only if every vertex is visible, and so an immediate consequence of Lemma 6.6 is that tree-child networks are labellable.

A phylogenetic network is *tree-sibling* if every vertex has a sibling that is a tree vertex (Cardona et al. 2008). In other words, every set of children (of a vertex in the network) has at least one tree vertex. Since a tree vertex is the child of exactly one parent, it is impossible for two sets of children to have the same parents, in a tree-sibling network. Thus, as an immediate corollary to Theorem 3.3, tree-sibling networks are also labellable.

### Corollary 6.7

(Corollary to Theorem 3.3) Tree-sibling networks are labellable.

Thus, the classes of orchard and tree-sibling networks are labellable, and other prominent classes, namely the tree-child networks and normal networks (Willson 2010), are both orchard and tree-sibling networks, and thus sit in their intersection (in particular, they are also labellable). The above findings are summarised in Fig. 6.

## Derived Networks

In this section, we return to the wider generality of allowing degenerate networks, and we show that every phylogenetic network has a quotient that is labellable.

Take any network $N=(V,A)$ with the leaf set [*n*] and define a relation $\sim$ on *V* by $x \sim y$ if and only if $c_N(x)=c_N(y)$. Then $\sim$ is an equivalence relation, and so *N* determines an associated network $N'=(V', A')$ where $V'$ is the set of equivalence classes of *V* under $\sim$, and $(u,v) \in A'$ if and only if there exists $x \in u$ and $y \in v$ with $(x,y) \in A$. Note that $N'$ has leaf set [*n*] and a single root.

In general, the network $N'$ may still have distinct vertices *u* and *v* for which $c_{N'}(u) \ne c_{N'}(v)$, in which case $N'$ is not labellable. Nevertheless, we can repeat the above process to construct a sequence $N, N', N'', \dots$ which stabilises after a finite number of steps at a network $\mathcal D(N)$ that has no distinct vertices *u*, *v* with $c_{\mathcal D(N)}(u)=c_{\mathcal D(N)}(v)$, and thus $\mathcal D(N)$ is labellable. Note that the number of steps required to reach this labellable network is finite, since replacing an equivalence class (of vertices) of size greater than one by a single vertex reduces the number of vertices in the network.

**Fig. 7:**
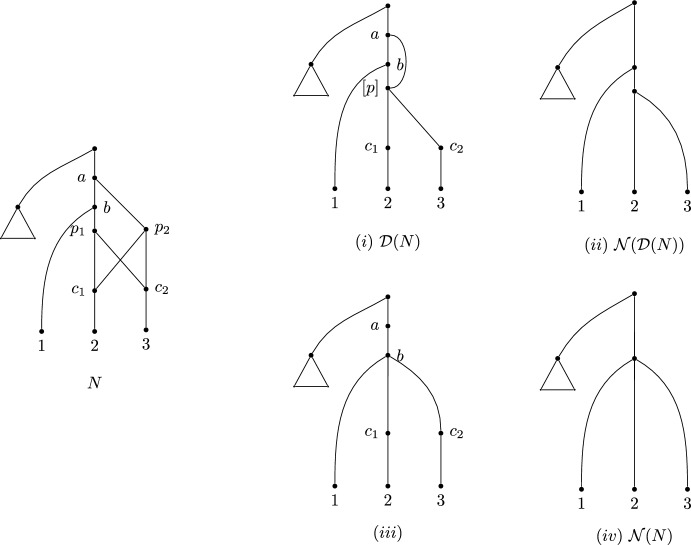
An unlabellable network *N* on the left, with the results of normalising, and taking the derived network. The pendant triangle represents an arbitrary tree. (*i*) shows its derived network $\mathcal D(N)$, where [*p*] denotes the $\sim$ equivalence class $\{p_1, p_2\}$, and (*ii*) shows the normalisation of that derived network, $\mathcal N(\mathcal D(N))$. The bottom row shows the normalisation process described in Francis et al. (2021). (*iii*) shows the visible vertices and related edges (the first step of the normalisation process), and (*iv*) shows the complete normalised network $\mathcal N(N)$ with degree-two vertices suppressed. Note that $\mathcal N(N)\ne \mathcal N(\mathcal D(N))$ for this example

This provides a canonical (and idempotent) map from all networks to the class of labellable networks. An example of this map is given in Fig. 7(ii).

### Connection to Normalisation

The derivation quotient contains an echo of the normalisation map in Francis et al. (2021), in that it is a map from the class of all phylogenetic networks to a specific class, in this case the labellable networks.

Recall that we write $\mathcal D(N)$ for the derived network of any network *N*, and let us write $\mathcal N(N)$ for its normalisation. The normalisation of a network *N* is obtained by taking all visible vertices in *N*, constructing the Hasse diagram of those vertices with the partial order defined by paths in the original network, and then suppressing any degree-two vertices. The result is a canonical normal network $\mathcal N(N)$ obtained from *N* (see Fig. 7 for an example, and (Francis et al. 2021) for further details). Note that normal networks are already labellable (since they are orchard, and by Theorem 6.4), so

$$\begin{aligned}\mathcal D(\mathcal N(N))=\mathcal N(N).\end{aligned}$$

On the other hand, the normalisation of a network and that of its labellable (derived) version need not be the same. That is, it is possible that

$$\begin{aligned}\mathcal N(\mathcal D(N))\ne \mathcal N(N).\end{aligned}$$

Thus, we obtain two normalisations of the same network, one via a labellization process. An example to illustrate this inequality is shown in Fig. 7.

## Discussion

We have described a combinatorial correspondence for phylogenetic networks that maps to sets of covers of a finite set. Covers of finite sets that grow in a constrained way (the expanding covers) correspond to the large class of labellable phylogenetic networks. The expanding covers that can be ordered in a particular way correspond to non-degenerate networks. The new class of labellable phylogenetic networks contains the class of orchard networks, but neither contains, nor is contained in, the class of tree-based networks. Because the class of labellable networks contains the orchard networks, it also contains the class of tree-child networks, and the class of normal networks.

There are a number of interesting further questions that remain to investigate.

For instance, we can describe when a *binary tree-based* network is labellable, based on the structure (Theorem 6.3). However, are there conditions on the covers that force the network to be *orchard*, or *tree-child*, or *normal*?

It would also be interesting to understand the link between covers from degenerate and non-degenerate networks more deeply. A degenerate network can be made non-degenerate in a simple manner: we suppress any degree-two vertices and blow up the vertices of in-degree and out-degree more than 1 by replacing each such vertex by two vertices connected by an edge (so that a vertex of in-degree $d_1$ and out-degree $d_2$ is replaced by one vertex of in-degree $d_1$ and out-degree 1, connected to another of in-degree 1 and out-degree $d_2$). What does removing the degeneracy mean for a cover, and what do these two degeneracy-removing actions involve at the cover level?

The connection between the removal of degeneracy and covers extends to other actions on networks. Is it possible to describe actions such as nearest neighbour interchange or subtree prune and regraft (SPR) in terms of changes to the cover? Indeed this can be asked in the context of familiar families of networks: can the cover of a network be manipulated to make it orchard, tree-child, or normal?

Finally, there may be connections to algebraic structures to explore. In the case of trees and forests, which correspond to partitions of finite sets, there is a corresponding set of partition diagrams that can be acted on by the elements of the symmetric group or Francis and Jarvis (2022). What, if any, are the corresponding algebraic structures that correspond to covers, and can these be used to move around the network space?

## Data Availability

Data sharing is not applicable to this article as no datasets were generated or analysed during the current study.
